# Synthetic and Natural Antifungal Substances in Cereal Grain Protection: A Review of Bright and Dark Sides

**DOI:** 10.3390/molecules29163780

**Published:** 2024-08-09

**Authors:** Tomasz Szczygieł, Anna Koziróg, Anna Otlewska

**Affiliations:** 1 Institute of Fermentation Technology and Microbiology, Faculty of Biotechnology and Food Sciences, Lodz University of Technology, 90-530 Lodz, Poland; tomasz.szczygiel@dokt.p.lodz.pl (T.S.); anna.otlewska@p.lodz.pl (A.O.); 2 Interdisciplinary Doctoral School, Lodz University of Technology, 90-530 Lodz, Poland

**Keywords:** chemical fungicides, biocontrol agents, integrated control, seed preservation

## Abstract

Molds pose a severe challenge to agriculture because they cause very large crop losses. For this reason, synthetic fungicides have been used for a long time. Without adequate protection against pests and various pathogens, crop losses could be as high as 30–40%. However, concerns mainly about the environmental impact of synthetic antifungals and human health risk have prompted a search for natural alternatives. But do natural remedies only have advantages? This article reviews the current state of knowledge on the use of antifungal substances in agriculture to protect seeds against phytopathogens. The advantages and disadvantages of using both synthetic and natural fungicides to protect cereal grains were discussed, indicating specific examples and mechanisms of action. The possibilities of an integrated control approach, combining cultural, biological, and chemical methods are described, constituting a holistic strategy for sustainable mold management in the grain industry.

## 1. Introduction

Molds are one of the most difficult pathogenic microorganisms to combat during crop production, from the sowing stage through the development of young plants to harvesting and storage. Grain can become infected with fungi in the field when the plant is ripening. The infection may come from the mother plant or from external sources, such as insects or wind. Commonly isolated genera belonging to field molds include *Fusarium*, *Alternaria*, and *Cladosporium*. These genera cause damage or death to embryos, are potential producers of mycotoxins, and contribute to disease in new seedlings if infected seed is selected for sowing. Storage molds are genera that invade seeds during storage in warehouses. They include, in particular, the genera *Aspergillus* and *Penicillium*. These genera reduce the germination capacity of grain, produce mycotoxins, and at high humidity contribute to the spontaneous heating of stored grains, which drastically reduces the quality of the seed material. Fungi can also attack grain immediately after sowing, sticking to the surface or entering into the seed tissue. They then cause the weakening or death of embryos, which contributes to large crop losses at the very beginning of the cultivation process. Infection of crops leads not only to serious crop losses but, in the case of mycotoxin accumulation also causes health problems in humans and animals.

There are several ways to protect plants from fungal attack. One of the most effective methods is seed dressing. Seed dressing protects the plant from the earliest stages of growth, which often translates into fewer mold infections in the adult plant. For this reason, plant protection products have been used in agriculture for many years. However, most conventional plant protection products contain high levels of synthetic chemical compounds, which may be toxic. To protect the environment and human health, the use of synthetic compounds is increasingly limited. For example, in 2020, European Union countries adopted a “farm to fork” strategy aimed at promoting sustainable agriculture and healthy food. This strategy has contributed to restricting the substances that can be used on crops. As more and more chemical compounds have been withdrawn from European markets, new more natural plant protection products that fit into the green deal strategy are being sought. Microbiological biopreparations offer one possible alternative to chemical plant protection products.

This article reviews the current state of the art regarding the use of antifungal substances in agriculture for protecting seeds against phytopathogens. It discusses the benefits and disadvantages of both synthetic and natural fungicides for cereal grain protection.

## 2. Bibliometric Data Mapping and Clustering

Bibliographic records on chemical and natural fungicides used for the protection of cereal grains were retrieved from the Scopus database. The following reference word combinations were used to search for relevant bibliographic records based on keywords, titles, and abstracts: ‘fungicides AND cereal AND grains’ and ‘biocontrol AND cereal AND grains’. The AND hyphen between reference words was used to search only for publications related to the described scientific area. No time period was indicated, but the oldest publication in the Scopus database for the combination of the words ‘fungicides AND cereal AND grains’ was from 1959, while the latest was from 2024 (data was searched in April 2024). In total, 349 records were found, 59 of which were published up to the year 2000, while 290 were published in the last 24 years. In the years 1963–1972, no records containing these keywords were found.

**Figure 1:**
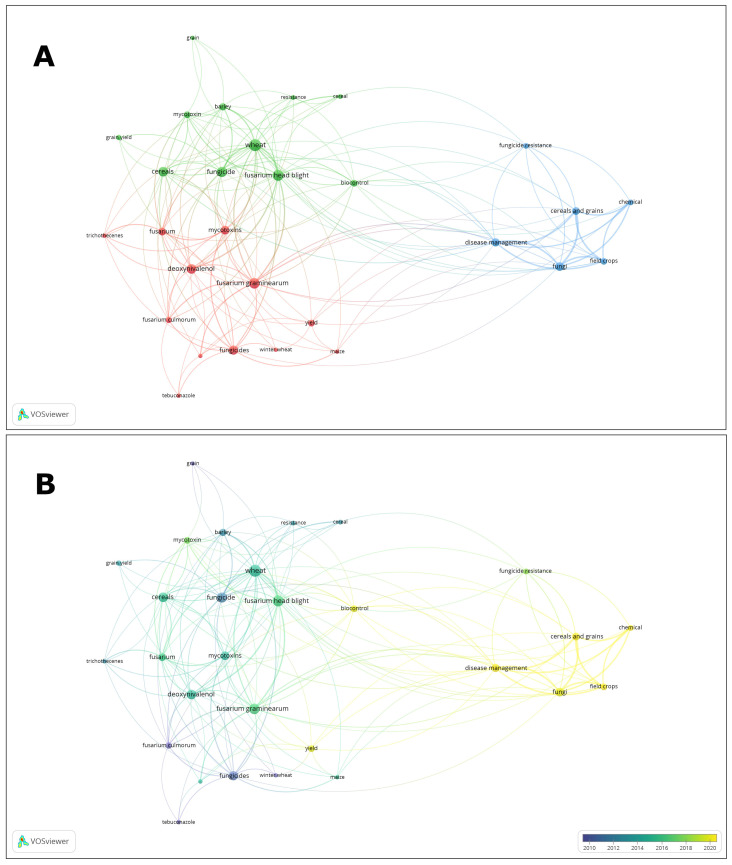
Term map based on keywords ‘fungicides’ and ‘cereal grains’. The colors on the map indicate terms belonging to different clusters (**A**) or the year of publication (**B**). The lengths of the lines correspond to the interrelationships between the terms. Bubble size presents the number of papers in the database. Bubble proximity presents frequency of co-occurrence of phrases in the same papers.

VOSviewer version 1.6.20 software (available at www.vosviewer.com, accessed on 30 May 2024) was used to visualize scientific landscape maps. VOSviewer distinguished three clusters, two of which were strongly related to the terms ‘biocontrol’ and ‘disease management’ (Figure 1A). It was in these areas that the publications from 2018 were focused (Figure 1B). The content of the papers included the mechanisms of fungicide action, based on information provided by the Fungicide Resistance Action Committee (FRAC). The advantages and disadvantages of synthetic agents and natural solutions were also discussed, including their impact on the environment, together with the possibility of simultaneous use of synthetic and natural substances using Integrated Pest and Disease Management (IDPM) strategies.

**Figure 2:**
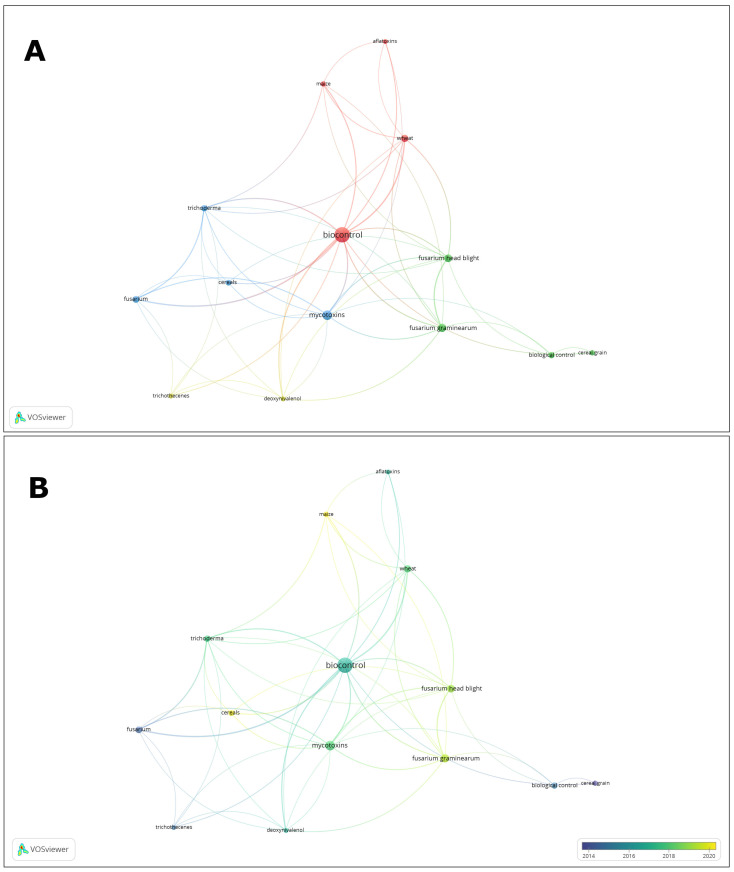
Term map based on keywords ‘biocontrol’ and ‘cereal grains’. The colors on the map indicate terms belonging to different clusters (**A**) or the year of publication (**B**). The lengths of the lines correspond to the interrelationships between the terms. Bubble size presents the number of papers in the database. Bubble proximity presents frequency of co-occurrence of phrases in the same papers.

For the combination of the words ‘biocontrol AND cereal AND grains’, 156 documents were found. Interestingly, the first record was not published until 1998, and the number of publications increased after 2018. Despite the lower number of publications than were found for the keywords ‘fungicides’ and ‘cereal grains’, four clusters were distinguished, indicating strong connections between the terms ‘biocontrol’, ‘mycotoxins’, and ‘*Fusarium graminearum*’ (Figure 2A,B).

## 3. Synthetic Fungicides

Fungicides are chemical substances used to combat fungi that infect plants. In agriculture, they are used to protect field crops: cereals, vegetables, fruit, ornamental plants, as well as greenhouse crops. Their task is to eliminate or inhibit the development of fungi and their spores. Fungicides can help to combat fungal infections during the sowing stage and during plant growth, improving the quality and durability of cultivated crops, as well as increasing harvest efficiency [1,2,3,4,5].

### 3.1. Short History

Plant pathogens such as fungi are often associated with significant losses during crop production, in both pre- and post-harvest scenarios. Over the past 100 years, the use of fungicides has contributed to reducing infections significantly [6], which has led to increased agricultural production efficiency [7]. Until the 1940s, the substances most commonly used to control diseases such as blights, mildews, and blasts were inorganic compounds of copper, which had been known since the mid-1700s [8]. In the early 1920s, the idea of using organic mercury compounds as a seed treatment emerged [9]. However, due to the high toxicity of mercury, this conservation method was abandoned in the 1960s, as it posed a danger to human and animal health as well as to the environment [10]. Further research led to the development of synthetic organic compounds in the form of dithiocarbamates in 1934, which were soon introduced on the market. Over the next 30–40 years, other compounds including phthalimides, fentins, and guanidines, were introduced [11]. These multi-site contact fungicides were characterized by low specificity and were limited to protection at the surface [12]. In the 1970s, the first systemic fungicide, carboxin (carboxamides), appeared, later followed by benzimidazoles, thiophanates, and morpholines [13]. In contrast to multi-site fungicides, systemic fungicides are site-specific, act internally to hinder infections, and can be used at much lower concentrations [7]. Since the turn of the 21st century, many new classes of fungicides have been developed. The most widely used are demethylation inhibitors (DMI), succinate dehydrogenase inhibitors (SDHI), carboxylic acid amides (CAA), and strobilurins [14]. These substances are most often paired with multi-site inhibitors to prevent pathogens from developing resistance due to the high specificity of the new fungicides [7]. Other recent approaches involve the use of compounds that activate the host’s defense system, such as physcion, probenazole, acibenzolar-S methyl, or dichlobentiazox [15,16].

Research on new products is driven by many factors, such as the development of resistance to previous generations of active substances, changes in legislation regarding the requirements for products, and demand from farmers themselves [17,18]. The type of substance used, the strain of the pathogen, and the method of applying the protective agents each have a large impact on the development of resistance [19,20,21]. Many of the fungicides currently in use are partially ineffective due to overuse or incorrect use [22]. For example, excessive use of triazoles causes the rapid development of resistance, the mechanism of action of strobilurins can be easily ignored by fungal cells that develop additional electron transport pathways, and the misuse of morpholines can lead to the development of fungal resistance. Specifically, morpholines inhibit the synthesis of ergosterol, an essential component of fungal cell membranes, by blocking the reduction reaction in the sterol biosynthesis pathway [1,19,23]. As well as pairing different substances, many other procedures are also used to extend the life (protective capacity) of these products as much as possible. These methods include the use of crop rotation, intercropping, or covering crops [24]. There is also legislative pressure to find innovative solutions. Directives from the European Union clearly indicate the parameters that should be characteristic of products intended for use in the EU, placing particular emphasis on their toxicological aspects in relation to both the environment and humans [25]. It is also important to remember that the substances used should have as negligible an impact on the plant as possible, so as not to cause additional yield losses. Despite this, some permitted substances can cause reduced transpiration, CO_2_ assimilation, and decreased production of biomass or sterols [1].

### 3.2. Cereal Crops around the Globe

Crops cover about 47.5 million square kilometers of the globe’s surface. Without adequate protection against pests and various pathogens, crop losses could be as high as 30–40% [26,27], which would most likely cause famine in many regions on earth. Various pesticides are used to prevent crop losses from occurring, including herbicides, insecticides, and fungicides. In 2022, it was reported that 117.9 kg/ha of pesticides were used for protective purposes in agriculture, of which 40% were fungicides. In the four examined countries/regions (Table 1) of the world, the EU had the highest usage of pesticides at 38.8 kg/ha, of which 19.8 kg/ha were fungicides. In the other countries/regions, pesticide use was notably lower [28]. According to FAO data from 2022 concerning the production of selected cereals (wheat, rye, barley, oats, and corn), China is the largest overall producer of corn (277.4 Mt), wheat (137.7 Mt), rye (0.5 Mt), barley (2 Mt), and oats (0.6 Mt), both in terms of the amounts produced and the area of cultivation. The USA produces similar amounts to China of rye, barley, and oats, but three times less wheat (44.9 Mt). Data collected from 27 EU countries show the highest values for cereal production related to wheat (134.3 Mt), barley (52 Mt), rye (7.5 Mt), and oats (7.5 Mt). Corn production in the EU (27 countries) is incomparably lower than in the other mentioned countries/regions, at 8.8 Mt. In Brazil, the quantities of cereals produced range from 10.3–11.2 Mt for wheat and rye and 0.5–1.6 Mt for barley and oats, while corn reached 109.4 Mt. China produced the largest quantity of wheat, at 137.7 Mt. The EU (27 countries) produced the largest quantities of rye (7.5 Mt), barley (52 Mt), and oats (7.5 Mt). The USA produced the most corn (348.8 Mt). Table 1 presents a summary of data on the annual production of cereals by the largest food producers in the world, with information about the cultivation area and the use of pesticides [29].

### 3.3. Areas of Fungicide Action in Mold Cells

**Figure 3:**
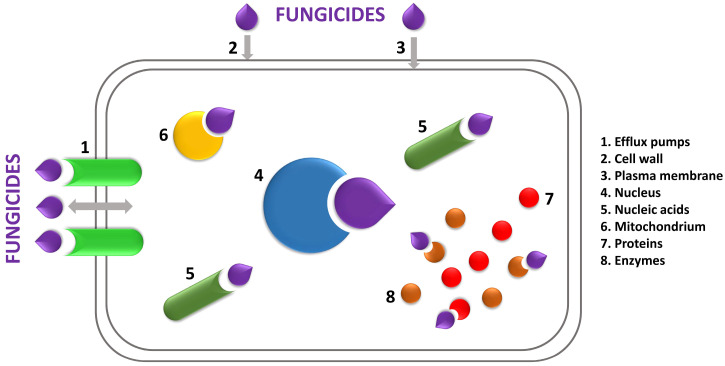
Fungicide mechanisms of action in mold cells.

There are several main areas of action by antifungal preparations, including fungicides, in fungal cells (Figure 3). The outermost structure is the cell wall, which is the first place of contact between biocidal substances and the cell. The cell wall is responsible for the osmotic balance in the cell and controls the secretion and uptake of molecules into the cell. There are many enzymes and other substances in the cell wall, including color compounds (melanin). In most species of fungi, over 80% of the dry weight of the cell wall consists of polysaccharides, including chitin, glucan, and mannan. Another area in the cell affected by biocidal compounds is the cell membrane. Its main function is to control the permeability of various types of particles, including phospholipids, sphingolipids, sterols, and proteins. A characteristic sterol component in fungi is ergosterol, which is also found in mitochondrial membranes. Ergosterol plays an important role in the cell membrane because it influences its stiffness and thus its permeability. If the fungicide penetrates the cell wall and membrane, it reaches the interior of the cell, where it may interfere with DNA and protein synthesis or affect respiration. Mitosis and cell division may also be the targets of fungicides [1,32,33,34].

Information on the mechanisms and areas of action of individual groups of fungicide compounds can be found on the websites of the Fungicide Resistance Action Committee (FRAC). This committee confers on fungicides a special FRAC code, which allows them to be properly grouped. The classification applies to the biochemical mode of action in cell plant pathogens. Active substances can be targeted at a specific structure or enzyme, inhibiting a single metabolic process, or have a multidirectional range of action. The alternating use of different classes of protection agents with different mechanisms of action can prevent the development of fungal resistance [35]. It should be noted that the division of fungicides into FRAC groups does not always coincide with the division taking into account the chemical structure of the compounds. An example is benzimidazoles—a chemical group, but in the FRAC system they belong to Group 1 (MBC—mitosis inhibitors) along with other chemical compounds such as thiophanates [35].

### 3.4. Fungicide Groups According to Their Mode of Action (MOA) in the Biosynthetic Pathways of Plant Pathogens

An attractive target for antifungal agents is chitin, which is an integral component of the cell wall. The key enzyme catalyzing chitin biosynthesis is chitin synthase. An example of a fungicide that inhibits the action of this enzyme is polyoxin, which belongs to the 19 FRAC group [36,37]. The cell wall also contains melanin, a multifunctional pigment that allows fungi to survive in unfavorable conditions. Melanin not only protects the cell against environmental stress and UV light but also plays an important role in pathogenesis. Several enzymes are needed for the synthesis of melanin, such as reductase, dehydratase, and polyketide synthase. According to the divisions introduced by FRAC, we distinguish several groups acting on the biosynthesis of this pigment:

- MBI-R (Melanin Biosynthesis Inhibitors—Reductase; 16.1 FRAC group) hydroxynaphthalene reductase inhibitors;
- MBI-D (Melanin Biosynthesis Inhibitors—Dehydratase; 16.2 FRAC group) inhibiting scytalone dehydratase;
- MBI-P (Melanin Biosynthesis Inhibitors—Polyketide synthase; 16.3 FRAC group) interfering with the activity of polyketide synthase.

All three types of fungicides can inhibit the production of melanin in fungal cells and, consequently, make them susceptible to unfavorable environmental conditions [38,39,40,41,42].

Another area of action of fungicides is the cell membrane, which consists largely of sterols and lipids. Lipid molecules are responsible for many biological functions in the fungal cell. They are an important reservoir of energy and are also part of the basic skeleton of the cell membrane. Specific proteins or enzymes needed for lipid metabolism therefore become the target of fungicides. The fungicide AH (Aromatic Hydrocarbons; 14 FRAC group) etridiazole hydrolyzes phospholipids of the cell membrane to produce lysophosphatides and free fatty acids, which in turn leads to the breakdown of membranes (lipid peroxidation). Another fungicide, AH dicloran, is a phototoxic compound that makes fungal membranes susceptible to sunlight, leading to the destruction of the linoleic acid structure. Dithiolates and phosphorothiolates (FRAC group 6) interfere with the biosynthesis of phospholipids by inhibiting the action of methyltransferases that catalyze methylation reactions. Carbamates (FRAC group 28) disrupt the permeability of the cell membrane, while plant extracts (FRAC group 46), such as tea tree extract (*Melaleuca Alternifolia*), cause disruption of the cell membrane. Polyene macrolides (FRAC group 48) bind ergosterol in the fungal cell membrane, which leads to the formation of hydrophilic channels. They change the permeability of the membrane, causing the outflow of intracellular contents. OSBPI (Oxysterol binding protein homolog inhibition; 49 FRAC group) fungicides inhibit the homolog of oxysterol binding protein (OSBP), which participates in the movement of lipid molecules between membranes. Inhibition of OSBP may also interfere with the maintenance of cell membranes and the formation of complex lipids necessary for cell survival [35,43,44].

A large group of fungicides are sterol biosynthesis inhibitors (SBI), which are capable of inhibiting various enzymes in the sterol biosynthesis pathway. Four classes of fungicides affecting the biosynthesis of sterols in the membrane can be distinguished: 1 SBI (DMI fungicides, demethylation inhibitors; 3 FRAC group), 2 SBI (amines, morpholines; 5 FRAC group), 3 SBI (KRI fungicides, ketoreductase inhibitors; 17 group FRAC), and 4 (18 FRAC group) [35]. DMI fungicides inhibit lanosterol 14α-demethylase, which is cytochrome P450 dependent. Morpholines lead to the inhibition of sterol Δ8, Δ7-isomerase, and sterol Δ14 reductase. Ketoreductase inhibitors inhibit 3-keto reductase and C4 demethylation. However, the 4th class of SBI fungicides, including e.g., naftifine and pyributicarb, is capable of inhibiting squalene epoxidase. Inhibition of even one of the enzymes necessary for sterol synthesis in the cell membrane leads to cell dysfunction and lysis [45,46].

An important structure in eukaryotic cells is the cytoskeleton, thanks to which the organelles do not float freely in the cytosol, but occupy certain places assigned to them. The polymers of the cytoskeleton are microtubules, which play an essential role in the cellular functions of fungi, e.g., cell division. They are therefore an important site for the action of biocidal compounds. An example is fungicides from FRAC group—MBC (Methyl Benzimidazole Carbamates) bind to the β-tubulin subunit in microtubules. This action inhibits the formation and proliferation of microtubules. By inhibiting mitosis, MBCs prevent the proper course of the fungal cell division process, resulting in its death. Microtubule polymerization inhibitors also include N-phenylcarbamates (FRAC group 10) and benzamide and thiazole carboxamide (FRAC group 22).

It is known that mycelium growth takes place in the top (apical) part of the hyphae. The stability of the membrane during elongation, especially at the hyphal tips, is determined by spectrin-like proteins located in this area. Their delocalization, caused by e.g., benzamides (FRAC group 43), results in inhibition of fungal growth. Other fungicides that affect the proper functioning of the cell are cyanoacrylates (FRAC group 47) and arylphenyl ketones (FRAC group 50). These probably interfere with the pathway that is responsible for regulating the actin cytoskeleton. They may also disturb the maintenance of cell polarity and hyphal morphogenesis, as well as inhibit the growth of polarized hyphae [47,48,49,50].

Fungi are aerobic organisms, and an important energy generator necessary for respiration are mitochondria. Mitochondria are the main regulator of the cellular metabolism of fungi and are the place where many physiological functions of the cell are coordinated. Hence, a FRAC group 39 of fungicides was created that inhibits the respiration of fungi. It consists of substances such as quinazoline, pyrimidines, and pyrazole-MET1, which act by influencing the complex I system of fungal mitochondria, inhibiting NADH oxidoreductases. SDHI fungicides (succinate dehydrogenase inhibitors; FRAC group 7) are complex II inhibitors that lead to dysfunction of the succinate dehydrogenase (SDH) enzyme in the mitochondrial electron transport chain and the Krebs cycle.

FRAC groups 11, 21, and 45 fungicides block electron transfer in mitochondrial complex III in cytochrome bc1. The first group of Quinone outside inhibitors (QoI) is capable of binding to cytochrome bc1 in the external quinoloxidizing pocket. Quinone inside inhibitors (QiI) bind in the internal quinone-reducing pocket. Quinone outside inhibitor stigmatellin binding type (QoSI) binds on the “outside” of the quinone in the stigmatellin binding subsite.

Fungicides belonging to the 29 FRAC group have an uncoupling effect on oxidative phosphorylation, which inhibits ATP production and interrupts the cellular metabolism of fungi. Organotin compounds (30 FRAC group) are inhibitors of oxidative phosphorylation and inhibit the action of ATP synthase. Thiophenecarboxamides (38 FRAC group) inhibit ATP transport [51,52].

The main building blocks of fungi are proteins consisting of amino acids. These proteins catalyze many biochemical reactions and participate in the formation of the cytoskeleton. Therefore, like sterols, proteins are an important cell component that can be targeted by the mechanisms of action of fungicides. According to the division into individual FRAC groups, we distinguish the following fungicides acting on proteins and amino acids:

- AP (Anilino-Pyrimidines; FRAC group 9), which is capable of secreting hydrolases and inhibiting methionine biosynthesis;
- enopyranuronic acid antibiotic (FRAC group 23), the active ingredient of which is blasticidin-S, which is capable of inhibiting protein biosynthesis by affecting ribosomal peptidyl transfer;
- kasugamycin, the active ingredient of hexopyranosyl antibiotic (24 FRAC group, which inhibits translation initiation and thus inhibits protein synthesis;
- glucopyranosyl antibiotics (FRAC group 25), which inhibit the initiation stage;
- tetracycline antibiotics (FRAC group 41), which are elongation inhibitors in protein synthesis and act on ribosomes [35,53].

Another important process that takes place in fungal cells is signal transduction—i.e., a number of biochemical processes (signals) originating from outside the cell or from its interior, leading to changes in life processes in the cell. Signal transduction in the cell occurs at the level of the cell membrane and is related to the functions of certain proteins. Abnormalities in signal transmission may result in cell dysfunction and, consequently, lead to cell death. An example are phenylpyrroles (FRAC group 12), which interfere with the osmoregulatory signal transmission pathway, interfering with the os-2 MAP kinase pathway. In germinating spores, they stimulate the accumulation of glycerol, which leads to cell swelling and, consequently, its rupture. Dicarboximides (FRAC group 2) also act on the osmotic signal transduction pathway and interfere with the os-1 MAP kinase pathway. As a result of cutting off signal transmission, they can inhibit the synthesis of glycerol as well as the development of mycelial hyphae. Aza-naphthalenes (FRAC group 13) also influence signal transduction, but their mechanism of action is not yet known [35,44,54].

The last group of fungicides categorized according to their mechanism of action against plant pathogens are fungicides that affect the synthesis of nucleic acids. PA fungicides (PhenylAmides; FRAC group 4) interfere with the synthesis of nucleic acids by inhibiting the activity of the RNA polymerase I system. Hydroxy-(2-amino-)pyrimidines (FRAC group 8) are responsible for inhibiting the action of the adenosine deamination hydrolyzing enzyme. This results in disruption of inosine production and, consequently, inhibition of nucleic acid synthesis. Carboxylic acids (FRAC group 31) interfere with the action of topoisomerases necessary for the functioning of the cell. They are used as inhibitors of type II DNA topoisomerase directed at gyrase. In turn, heteroaromatic fungicides (FRAC group 32) interfere with DNA/RNA synthesis in the fungal cell [55,56].

It can be concluded that fungicides are a very wide group of compounds. They can influence plant pathogens in various ways, acting on many areas, processes, and individual chemical compounds in their cells to prevent fungal diseases from damaging and destroying plants.

### 3.5. Fungicides in Crop Protection

Fungal diseases present one of the main threats to crops, so the use of fungicides is often necessary to secure global food supplies. Due to their ease of use, effectiveness in combating fungal plant diseases, and relatively low cost compared to possible losses, the use of fungicides has become very common in agriculture [1,57]. Fungicides can be divided according to several criteria:

A Stage of application:
  - Preventive: These fungicides protect plants by preemptively guarding against pathogenic attacks;
  - Interventional: Applied immediately after the onset of infection, these fungicides aim to halt further proliferation of the pathogen;
  - Destructive: Designed to eradicate spores and mycelium, thereby curbing the spread of the disease.
B Effect of active substances of plants:
  - Contact: These fungicides act on the plant’s surface, inhibiting spore germination and other vital processes;
  - Translaminar: This type of fungicide penetrates and moves within the leaf tissue, providing protection throughout the leaf structure;
  - Systemic: These fungicides penetrate plant tissues and distribute within them, creating a barrier against infection and fungal growth;
  - Local: These fungicides exert their effects by penetrating only a few cell layers at the site of application
C Methods/places of application:
  - Seed dressing: Application of finely ground solid particles dusted onto the seeds surface;
  - Foliar fungicides: Sprayed onto the foliage of plants at different growth stages;
  - Root/soil application: Soil drenching or injection into the soil underneath it near the roots.
D Type of active substance used in fungicides.
E Mechanisms of action of active substances on fungal cells.

Fungicides for agricultural crops can be used for both preventive and destructive purposes. Examples of the preventive use of fungicides include seed treatment, soil drenching, and spraying during the early developmental stages of plants. If preventative measures fail, destructive measures are used to manage the spores and mycelium. Most often, this involves spraying the plants with fungicide, as this allows for the fastest control of infectious foci [58].

There are many forms of fungicides currently available on the market, including dusts, gases, granules, and the most common form, which is liquid. Seed treatment fungicides can be applied in either wet or dry forms, depending on the type of seed. Powdering is a dry method used in seed protection, whereas wet methods include the usage of water dispersible powders, liquid solutions, and flowable solutions [7,24,59,60]. Fungicides can also be used during soil preparation and plant growth. When used on plants, the most efficient method is spraying, which can be performed as needed. Most often, foliar fungicides are applied on young plants in the preliminary stages of development to avoid infection. If infection is already present, the fungicide is used to eradicate fungal corruption and ensure that the pathogens will not be carried over into later developmental stages. The purpose of post-harvest use of fungicides is mostly to protect the harvested material against pathogens during storage [1]. Examples of fungicides used for seed treatment include captan, difenoconazole, and iprodione. Iprodione, as well as tebuconazole and pyraclostrobin, can also be used for foliar spraying. A more complete list of the most common fungicides for plant protection currently in use or recently phased out by large crop producers is given in Table 2. Of the 43 fungicides presented in the table, 27 are banned in the EU, 6 are banned in the USA, 11 are banned in China, and 9 are banned in Brazil. The groups with the most active substances include DMIs fungicides (demethylation inhibitors, 3 FRAC group)—11 substances; QoI (quinone outside inhibitors, 11 FRAC group)—5 substances; MBC (methyl benzimidazole carbamates, 1 FRAC group)—4 substances. The fungicide groups with the highest overall usage include dithiocarbamates (FRAC group M 03), DMIs (Demethylation Inhibitors, FRAC group 3), and QoIs (Quinone outside Inhibitors, FRAC group 11), especially strobilurins [57].

Until recently, there was a large range of plant protection products available. However, due to more restrictive legal regulations, such as Regulation EC 1107/2009 [63] and Directive 2009/128/EC [123], many products have already been withdrawn or will be withdrawn soon. Substances withdrawn from circulation in the EU in 2020–2022 included frequently used fungicides such as benalaxyl, epoxiconazole, thiophanate methyl, triflumizole, carboxin, cyproconazole, diethofencarb, fenbuconazole, fluquinconazole, flutriafol, mancozeb, prochloraz, and triazoxide. In 2023, four fungicides were banned, namely benthiavalicarb, dimoxystrobin, ipconazole, and metiram. Additionally, 34 important fungicides for crop protection were planned for reassessment (and eventual withdrawal), including fluazinam, pyraclostrobin, cyprodinil, fosetyl, metconazole, boscalid, captan, fluoxastrobin, prothioconazole, tebuconazole, penconazole, and tetraconazole [124,125,126,127,128] (Reg. 2023/114; Reg. 2023/689; Reg. 2023/918; Reg. 2023/1446; Reg. 2023/2592). However, all 34 compounds have since received extensions of approval for use [25,63]. By 1 January 2024, of 1477 active substances used in pesticides registered in the EU database, 951 had been banned. In the literature, there are currently 490 fungicidal substances known. Not all of these 490 substances were previously registered in the EU. After comparison of the substances present in the literature and EU database, 175 of these are banned. Within those 175 fungicides, 78 were used in agriculture, with 71 potentially used for seed treatment. Notably, common grain protection fungicides including mancozeb, prochloraz, propiconazole, and thiophanate-methyl were among those withdrawn.

### 3.6. Synthetic Antifungals and the Environment

**Figure 4:**
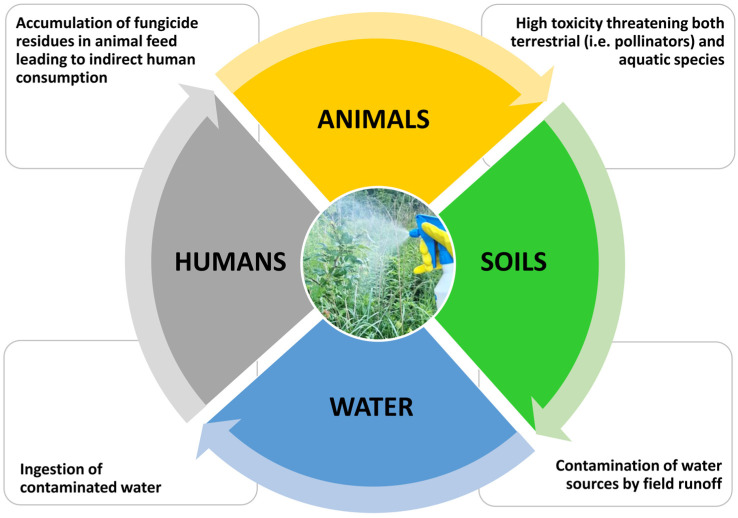
Fungicides and their impacts on various environmental zones.

Many synthetic fungicides have been or will be withdrawn in the near future, primarily due to their toxicity to humans and the environment (Figure 4) [24]. An example of the tragic effects of pesticide (including fungicide) use is the decline in the number of birds in the United Kingdom during the period 1967–97. Data show that between 23% and 89% of birds died out in this period, depending on the species. In addition, these compounds accumulate in the soil and contaminate water as they run off fields, posing a threat to microbial diversity for long periods of time, depending on the ability of the substance used to break down over time [129,130,131,132]. In aquatic environments and agricultural fields, excessive concentrations of pesticides may cause a breakdown of the balance of microorganisms present in the environment, or even affect the crop itself [133]. Beneficial insect species such as pollinators may also suffer. For these reasons, a new generation of fungicides is sought that will not have a negative impact on soil structure and biocenosis. These new solutions should have low toxicity and aggregation capacity to ensure the safety of both humans and the environment.

Many factors affect the decomposition of pesticides used in agriculture, including access to light, temperature, and the microbiome. Depending on the combination of these factors, the half-life of pesticides can range from a week to a year [134]. The long-term degradation of synthetic fungicides causes serious environmental pollution. Furthermore, misuse and overuse of fungicides can be harmful for non-target species occurring in the environment and disturb its natural biodiversity. The degradation of synthetic fungicides can also create problems beyond the agricultural industry. An example is the development of cross-resistance in clinical strains, which through interactions with resistant environmental strains become resistant to azole drugs used in medicine. *Aspergillus fumigatus* found both in fields and in medical cases of aspergillosis is one of the best-known instances of this problem [135].

However, synthetic fungicides also have clear benefits. Fungicides are able to control many pathogenic fungi, thanks to which they can be used both for protective and destructive purposes in the event of infection. They thereby play an important role in preventing crop losses and increasing food safety, which is crucial in the context of an ever-growing population [136].

It seems clear that chemical agents will continue to be used, and new synthetic fungicides will be needed as current solutions become less effective over time. However, given the disadvantages of chemical plant protection products, it seems rational to combine the use of synthetic fungicides with natural biofungicides [7,137].

## 4. Biological Methods of Cereal Preservation

Biological control agents (BCAs) are a group of living organisms including microorganisms, insects, mites, nematodes, protozoa, and viruses, as well as botanical (plant extracts) and semiochemical (pheromones, kairomones) substances used to protect plants and seeds from pests and phytopathogenic diseases [138]. Synthetic agrochemicals are excluded from the group of BCAs. However, several substances occurring in nature that are equivalents of synthetic substances (semiochemicals) are permitted [139,140]. Although the term biological control has existed for over 100 years (introduced by Smith in 1919), the terminology in this area probably shows divergences depending on whether it is formulated by entomologists or biologists. In 2001, Eilenberg et al. [141] and later in 2017 Heimpel and Mills [142], proposed a harmonized definition for all biological control areas, but some inconsistencies still exist. The entry into force of Regulation (EC) No 1107/2009 on plant protection products (PPPs) [63] resulted in an increase in the use of BCAs in Europe. This regulation does not clearly define BCAs, and there is still a lack of a universally accepted definition of biocontrol. This may result in confusion and misuse among researchers and potential users regarding the safe use of biopreparations [143].

BCA-based seed treatment technology includes tools to improve seed quality, primarily reducing damage caused by pathogens and rot diseases. Both factors can drastically affect the germination process and therefore the final crop yield. However, seeds have developed their own passive physical (reinforced cell walls) and chemical (antimicrobial compounds) mechanisms, which are dependent on genetic and environmental factors, in response to attack by organisms and phytopathogens. These mechanisms are activated during seed germination, or in some cases during the rehydration cycle in the soil. In the dormant state (e.g., during storage), seeds are unable to activate defense mechanisms, suggesting that this is an appropriate period to install exogenous barriers as a preemptive strategy to protect grains [144,145]. The treatment of the seed surface with exogenous active substances (liquid or solid, forming a more or less continuous layer) using a binder or, in some cases, a filler that can act as a carrier has been proposed as a precise and cost-effective method [146,147]. Various types of seed treatment are used to improve the functional properties of seeds, including seed dressing, coating, soaking, and granulating, depending on the type and purpose of the seeds or the type of active ingredients selected for inoculation (whole microbial cells, natural active compounds, etc.) [147]. Seed coatings were first used in the 1930s for cereal grains, although their large-scale commercial use did not occur until 30 years later [148]. Nowadays, seed dressings are widely used in the crop and horticultural industries around the world to apply dyes, pesticides, soil adjuvants, germination and growth stimulants, substances that increase resistance to stress (e.g., salicylic acid, gibberellic acid, and abscisic acid), macronutrients and microelements, as well as biological control agents. The coatings with BCAs have been successfully applied to many different sizes, shapes, textures, and germination types of seeds, especially cereal grains [147]. Therefore, here, we will mainly focus on natural antifungal substances employed for protecting cereal grains.

### 4.1. Bacteria, Yeast, and Molds as Biocontrol Agents

The use of microorganisms as BCAs to protect against cereal seed-borne diseases is based mainly on their ability to synthesize a wide range of secondary metabolites (e.g., antibiotics, hydrogen cyanide) and lytic enzymes that can inhibit the growth of phytopathogens, as well as the activation of induced systemic resistance [149,150]. After the end of 2022, four new regulations (EU Regulation 2022/1438, 2022/1439, 2022/1440, 2022/1441) approved in the EU entered into force to simplify the approval process and authorization of biological plant protection products containing microorganisms. Currently, 71 strains of microorganisms are approved in the EU; another 26 strains are in the process of being approved [143].

The main microbial biocontrol agents with strong antifungal activity are *Bacillus* spp., which produce fungicidal and fungistatic metabolites. Endophytic strains of *Bacillus subtilis*, *Bacillus velezensis*, *Bacillus inaquosorum*, and *Bacillus nakamurai* inhibit the growth of *Fusarium graminearum* (*Gibberella zeae*) and *F. poae* on various cereal grains (barley, wheat) both in vitro, in greenhouse and under field conditions [151,152,153]. Pan et al. (2015) demonstrated the ability of *B. subtilis* and *Bacillus megaterium* species to inhibit the growth and spore germination of *F. graminearum* [154]. *Bacillus vallismortis* has been reported to have a wide spectrum of antifungal activity [155]. The filtrate and extract of *B. vallismortis* were found to inhibit the growth of several phytopathogens, including *Fusarium graminearum*, *Alternaria alternata*, *Rhizoctonia solani*, *Cryphonectria parasitica*, and *Phytophthora capsici*. The strong antagonistic activity of these species results from the secretion of antifungal lipopeptides, namely bacillomycins, iturins, surfactins, fengycins, and mycosubtilins [153]. *Bacillus* spp. is also known to synthesize hydrolytic enzymes such as amylase, cellulase, chitinase, lipase, phytase, and protease, important in biocontrol processes. These spore-forming bacteria are characterized by a high degree of adaptation to environmental stress conditions, which allows them to easily inhabit various niches. Due to the rapid colonization and utilization of nutrients (carbon, hydrogen, oxygen, nitrogen) and microelements (iron, manganese, copper, zinc, phosphorus) by *Bacillus* spp., they make the environment less favorable for the development of pathogens [156].

Other promising biocontrol agents for the protection of grains against *Fusarium* spp. include cell-free supernatants of lactic acid bacteria *Lactiplantibacillus plantarum* and *Lacticaseibacillus rhamnosus* [157,158].

The multidirectional antifungal activity of *Streptomyces* spp. has been demonstrated in many studies [150]. These bacteria are known to synthesize various secondary metabolites (e.g., 2-(chloromethyl)-2-cyclopropyloxirane, 2,4-ditert-butylphenol and 1-ethylthio-3-methyl-1,3-butadiene) that can inhibit the development of phytopathogens. They also (e.g., *Streptomyces lydicus*, *S. mutabilis*) produce enzyme—chitinase that can degrade the cell walls of various phytopathogenic fungi such as *Fusarium* spp. and *Rhizoctonia solani* [150,159]. In addition to bioactive substances and enzymes, many *Streptomyces* (e.g., *Streptomyces platensis*, *S. noursei*, *S. fimicarius*, *S. albulus*) are producers of volatile organic compounds (VOCs, e.g., anisole, alpha-copaene, caryophyllene, ethyl phenylacetate, methyl anthranilate, methyl salicylate phenylethyl alcohol, 4-methoxystyrene, 2-pentylfuran, and 4-ethylphenol) with antimicrobial activity against phytopathogenic species *Botrytis cinerea*, *Rhizoctonia solani*, *Fusarium oxysporum*, and *Sclerotinia sclerotiorum* [150,160].

Biocontrol ability is widespread among yeast species. Matić et al. (2014) found that seed coating with *Pichia guilliermondii*, *Metschnikowia pulcherrima*, and *Sporidiobolus pararoseus* inhibited growth of *Fusarium fujikuroi* [161]. Druvefors and Schnürer (2005) investigated the antagonistic activity of 57 different yeasts belonging to the *Pichia* (*P. anomala*, *P. guillermondii*, *P. burtonii*, *P. farinose*, *P. membranifaciens*) and *Candida* (*C. silvicola*, *C. fennica*, *C. pelliculosa*, *C. silvicultrix*) genera against *Penicillium roqueforti*, one of the most important spoilage molds in airtight-stored cereal grains [162]. *Clonostachys rosea* (formerly named *Gliocladium roseum*) also shows promising biocontrol activity. To date, its antifungal activity has been documented against 11 mold genera, including *Fusarium*, *Alternaria*, and *Botrytis* (Table 3), which are associated with the secretion of the cell wall-degrading enzymes chitinase, glucanase, and protease [163]. Jensen et al. (2000) used both stored (dried) and fresh conidia of *Clonostachys rosea* to treat cereal grains infected with *Fusarium culmorum* in field conditions. They achieved more than 80% disease control with both types of conidia [164].

A particularly promising strain for reducing the population of fungal pathogens, especially on cereal grains, is *Trichoderma* spp. [175]. As stated by Benitez et al. (2004), 90% of fungal strains used to control plant diseases belong to the genus *Trichoderma*. The most known and described BCA species are *T. viride*, *T. virens*, *T. harzanium*, *T. hamatum*, *T. longibrachiatum*, *T. koningii*, *T. lixii*, *T. polysporum*, and *T. asperellum* [176,177,178]. The success of *Trichoderma* spp. as a BCA is related to its multidirectional and comprehensive mechanisms. On the one hand, *Trichoderma* inhibits the development of phytopathogens such as *Alternaria brassicicola*, *Armillaria mellea*, *Fusarium equiseti*, *F. guttiforme*, *F. graminearum*, *F. oxysporum*, *Phytophthora palmivora*, *Rhizoctonia solani*, *Sclerotium rolfsii*, and *Sclerotinia sclerotiorum* [178,179]; on the other hand, they can stimulate growth and plant defense mechanisms. The wide spectrum of antagonistic activity of *Trichoderma* spp. is related to a combination of mycoparasitism, antibiosis, secretion of enzymes, and competition for space and nutrients [175,176,177,178,179,180]. It is considered as a necrotrophic mycoparasite and obtains nutrients by penetrating the pathogen’s cell wall without causing host cell death. Moreover, these fungi secrete cellular enzymes including chitinase, protease, glucanase, cellulase, xylanase, pectinase, lipase, and amylase, leading to the penetration and degradation of cell walls and causing plasmolysis [173,175]. Over 90 metabolites (low-molecular-weight volatile or non-volatile compounds) have also been detected in *Trichoderma* spp. (e.g., trichorzianin TA, trichorzianin TB, trichodermin, tricholin, cyclonerodiol, pachybasin, 6-pentyl-2H-pyran-2-one) with antifungal activity [181]. Xue et al. (2017) showed the strong antifungal activity of six strains of *Trichoderma* spp. (*T. asperellum*, *T. citrinoviride*, *T. harzianum*) against *F. graminearum* in wheat [172]. The same authors also showed that treating wheat seeds with spores of the tested mold species (10^7^ spores/mL) reduced wheat root rot by >50%. Ferrigo et al. (2020) observed a 36% reduction in the occurrence of *F. verticillioides* and *F. graminearum* on grains coated with *Trichoderma harzanium* spores (10^5^ spores/mL) [174].

In addition to inhibiting/limiting the growth of phytopathogens, microorganisms applied to cereal grains may perform another important function: detoxifying mycotoxins produced by fungi contaminating the grains. This is extremely important from the point of view of the dangers posed by the presence of aflatoxin B1 (AFB1), ochratoxins (OTA), fumonisins (FUM), deoxynivalenol (DON), zearalenone (ZEN), and T2/HT-2 toxins (T2). The most frequently detected mycotoxins in grains include OTA (wheat, barley, maize), ZEN (wheat, maize), DON (wheat, barley, maize), AFB1 (maize), and T2 (wheat, rye, oat, maize) [182]. According to research conducted by BIOMIN on cereals and cereal products, the most widespread mycotoxins in the world are DON (66%), FUMs (56%), and ZEN (53%) [183].

The ability to degrade mycotoxins is mainly demonstrated by molds, followed by bacteria and, less frequently, yeasts. Decontamination strategies to reduce mycotoxins are based on biodegradation, bioadsorption, or inhibition of mycotoxin production [184]. Biological control of mycotoxins offers an excellent alternative to chemical and physical methods to protect food and feed and is currently of great interest to researchers and industry. However, biodegradation may result in the formation of more toxic compounds. Therefore, it is necessary to analyze the toxicity of the resulting compounds [185].

According to the literature, nontoxigenic fungi including *Aspergillus* spp., *Clonostachys* spp., *Penicillium* spp., *Rhizpous* spp., and *Trichoderma* spp. are active agents reducing mycotoxins. Most studies have been conducted in vitro or in a simple system reflecting field conditions, with few in vivo studies directly on cereal grains. The literature data indicate that the most effective genus of fungi is *Trichoderma*. Błaszczyk et al. (2017) investigated 24 fungal strains of *Trichoderma* spp. (including *T. atroviride*, *T. cremeum*, *T. hamatum*, *T. harzianum*, *T. longipile*, *T. viride*, *and T. viridescens*) for inhibition of growth and mycotoxin biosynthesis by *Fusarium* spp. [186]. *Trichoderma atroviride* was found to have the ability to reduce not only DON and ZEN (69–100%), but also nivalenol, beauvericin, and moniliformin. The mycotoxin reduction capacity of *Trichoderma atroviride* and *Trichoderma harzianum* has also been the subject of research by Tian et al. (2018) [187]. These authors suggest that the mechanism of ZEN reduction by *Trichoderma* spp. is based on sulphation, which leads to the formation of zearalenone sulphate zearalenol sulphate. *Trichoderma gamsii* has been found to be an effective suppressor (28–68% reduction) of fumonisin B1 and fumonisin B2 in maize seeds [188]. According to Dini et al. (2022), *Trichoderma afroharzianum* can produce various enzymes, including glucanases, chitinases, and cellulases active against phytopathogens, but also peroxidases able to degrade mycotoxins such as AFB1 and OTA [189].

Many bacteria have the ability to detoxify one or more types of mycotoxins. The degree of mycotoxin detoxification depends not only on the bacterial strain but also on the substrate, pH, and temperature. Lactic acid bacteria are able to remove mycotoxins by cell-surface binding [190]. Lactic acid bacteria capable of cell-binding mycotoxins mentioned in the literature include *Lactobacillus acidophilus*, *Lacticaseibacillus rhamnosus*, *Lactiplantibacillus plantarum*, *Lactobacillus amylovorus*, and *Limosilactobacillus fermentum.* The mycotoxin binding efficiency of the mentioned LAB ranges from 60% to even 98% [190,191,192]. *Streptomyces* spp. and *Bacillus* spp. can remove mycotoxins via biodegradation. Harkai et al. (2016) examined 124 strains of *Streptomyces* spp. and showed that *Streptomyces rimnosus* was effective at biodegradation of ZEN mycotoxin (88% degradation), while *Streptomyces cacaoi* totally degraded AFB1 mycotoxin [185]. *Bacillus* spp. also have the potential to degrade ZEN and AFB1. Tinyiro et al. (2011) and Farzaneh et al. (2012) analyzed strains of *B. subtilis*, *B. natto*, and *B. velezensis* both in liquid cultures and on the food matrix of pistachio nuts [193,194]. It has also been shown that cell-free supernatant of *B. subtilis* and *B. amyloliquefaciens* reduced mycotoxin synthesis by *A. parasiticus*, *A. niger*, and *Penicillium* spp., and remained stable in the temperature range from −20 °C to 100 °C [195]. Yang et al. (2017) studied the ability of *Pseudomonas fluorescens* to degrade AFB1, with peanut kernels used as a matrix [196]. Over 88% detoxification was achieved after 96 h.

Yeast can degrade mycotoxins via biodegradation and bioadsorption. The ability to reduce mycotoxins (AFB1, OTA, and ZEN) by cell-binding has been demonstrated in *Saccharomyces cerevisiae*, *S. uvarum*, *Kluyveromyces marxianus*, and *Candida utilis* [197]. In the case of *S. cerevisiae* yeast, this mechanism is probably related to the presence of (1,3)-D-glucans in the cell wall [190].

Microbial biocontrol agents are considered a sustainable tool for the protection of crops, including cereals. Biofungicides can limit/inhibit the growth of phytopathogens, reduce the mycotoxins they produce, stimulate plant germination and growth, as well as enhance soil ecosystem function. They are biodegradable, friendly to non-target species, and do not cause phytopathogens to acquire resistance. Compared to synthetic fungicides, they have less adverse impact on the environment, with a shorter residual effect [198,199]. Nonetheless, questions remain about the safety of some forms of biological control. Goettel et al. (2001) identify allergenicity, toxicity, and pathogenicity as possible harmful effects of fungi used as biocontrol agents [200]. It is common knowledge that some species of fungi can cause allergies. There are no direct reports that fungi used as biocontrol agents are responsible for the production of common allergens, but such a risk exists with mass production and application. Both fungi and bacteria secrete a wide spectrum of compounds, many of which may be crucial in the biocontrol process, but which may also be toxic or pathogenic to plants, invertebrates, and vertebrates. Although most strains of biofungicides meet all biosafety requirements, there are potential risks associated with using the species *Burkholderia cepacia*, *Pseudomonas putida*, *Pantoea agglomerans* and *Aureobasidium pullulans*, which are considered opportunistic human pathogens [201]. It is worth noting that *Trichoderma* spp. is on the ever-growing list of fungal pathogens, although it rarely causes infections in humans. The most common pathogen of this genus causing infection is the species *T. longibrachiatum*. Therefore, its use in agriculture, should be limited. All microbial strains intended for use in agriculture should also be tested for possible pathogenicity, to minimize the risk of spreading diseases [202].

Another issue is the impact of biofungicides on the autochthonous microbiome in the environment. Most biofungicides are tested only under laboratory conditions. As noted by Bonaterra et al. (2012), before employing them in agriculture, it is necessary to monitor the behavior of microbial strains used as biocontrol factors in the ecosystem [201]. This includes their establishment, adaptation, survival, dispersal, genetic and phenotypic stability, and the risk of horizontal gene transfer (e.g., antibiotic resistance). It is important to ensure that, as a result of adaptation, biofungicides will not dominate the environment, disturbing the natural biodiversity of the microbial community. This seems to be the most difficult challenge for ensuring the safety of using biofungicides. Regulations in many countries already require environmental impact analysis of biopesticides as part of the registration and commercial development process [201].

Biocontrol agents are biomolecules with a short shelf life that require special formulations to guarantee appropriate effectiveness and stability [145]. One of the most significant challenges related to the use of BCAs in agriculture (which is less important in the case of grain protection) is their adaptation to environments highly exposed to pollution (including agrochemicals) and to changing environmental conditions (high/low temperatures, drought, heavy rainfall). The advantage of BCAs is that they are easily degraded but do not have lasting effects in the environment.

### 4.2. Antifungal Activities of Plant Extracts and Essential Oils on Grains

Plant extracts and essential oils (EOs) extracted from plants constitute an eco-friendly alternative to synthetic pesticides used on cereal grains, legumes, fruits, and vegetables. Prakash et al. (2012) found that EOs contain a mixture of various major and minor elements that are responsible for their biological activity [203]. This means that the chance of developing resistant fungal strains is lower than in the case of many synthetic fungicides used in agriculture. Some EOs have been shown to have not only strong fungicidal properties against molds, but also a remarkable ability to reduce mycotoxin synthesis. Taheri et al. (2023) list the mechanisms of antifungal action by EOs as follows: inhibition of cell wall formation, inhibition of cell division and development of the mitotic spindle, inhibition of nucleic acids and protein synthesis, inhibition of efflux pumps, disruption of cell membrane function, and mitochondrial dehydrogenase function [204,205]. The biological activity of EOs is attributed to the presence of main components such as bioactive terpenes, alkaloids, and phenolic aromatic molecules [206]. It appears that EOs with high levels of phenolic compounds have stronger antifungal properties.

Numerous studies have investigated the antifungal activity of various plant extracts and EOs from basil (*Ocimum basilicum* L.), rosemary (*Rosmarinus officinalis* L.), thyme (*Thymus vulgaris* L.), marjoram (*Origanum majorana*), ginger (*Zingiber officinale*), oregano (*Origanum vulgare*), cinnamon (*Cinnamomum zeylanicum*), palmarosa (*Cymbopogon martinii*), lemongrass (*Cymbopogon citratus*), clove (*Eugenia caryophyllata*), garlic (*Allium sativum*), lavender (*Lavandula angustifolia* Mill.), mint (*Mentha piperita* L.), sage (*Salvia officinalis* L.), tansy (*Tanacetum vulgare* L.), yarrow (*Achillea millefolium* L.), and wormwood (*Artemisia absinthium* L.) against, among others *Aspergillus* spp., *Alternaria* spp., *Penicillium* spp., and *Fusarium* spp. ([207,208,209] and references therein). However, the conclusions are still debated, especially since most of the research has been conducted in vitro under laboratory conditions and not directly on cereal grains during storage.

One of the many methods of applying essential oils to cereal grains is fumigation. Anžlovar et al. (2017) reported the antifungal potential of thyme essential oils against a group of endophytic fungi from wheat seeds, namely *Alternaria alternata*, *Alternaria infectoria*, *Aspergillus flavus*, *Epicoccum nigrum*, and *Fusarium poae* [210]. In the case of grains, fumigation has proven to be more beneficial than soaking because the EOs significantly inhibit fungal growth, while soaking the grains in EOs inhibits their germination. The use of EO from laurel (*Laurus nobilis* L.) and laurel components as antifungal agents for wheat grains has been investigated by Belasli et al. (2020) [211]. The laurel EO showed high effectiveness (51.5–76.7%) in protecting fumigated wheat grains against *A. flavus* contamination during storage for 6 months. Lee et al. (2021) evaluated the antifungal activity of lemongrass EO in the form of soaked sachets against *Aspergillus flavus* on wheat seeds stored for 30 days [212]. After the storage period, the lemongrass EO showed a high fungicidal effect, reducing fungal growth by nearly 100%.

Fumigation with essential oils has proven to be an effective process against phytopathogenic fungi not only on wheat grains but also other seeds. Ben Miri et al. (2023) studied the effect of fumigation with two EOs and combinations of these EOs on maize, barley, and rice grains [206]. Menthol, eugenol, and a mixture of both reduced mycelial growth and spore germination of *A. ochraceus* and *A. niger* stored in closed containers by over 50%. Roselló et al. (2015) demonstrated 90–100% effectiveness of cinnamon, clove, and oregano oils (at concentrations of 200 μg/mL) against fungal growth on rice grains stored at 28 °C for 30 days [213]. Essential oil of oregano showed potential to control the common fungal phytopathogens *F. verticillioides* and *F. culmorum* on rice seeds. Bocate et al. (2021) used fumigation with garlic EO on maize [214]. The growth of fungi *Aspergillus parasiticus*, *Fusarium verticillioides*, and *Gibberella zeae* was completely inhibited with concentrations of the EO ranging from 2 µg/L to 10 µg/L. On maize grains contaminated with *Fusarium graminearum* and *Fusarium culmorum*, Perczak et al. (2019) verified the effectiveness of EOs from oregano herb, cinnamon bark, palmarosa leaves, orange peel, verbena leaves and flowers, spearmint leaves, rosewood, and fennel seeds [215]. All of the tested oils reduced fungal growth without negatively affecting seed germination.

Sustainable agriculture practices that reduce reliance on synthetic chemicals are vital for ensuring food security while safeguarding the environment and human health. Currently, farmers rely heavily on synthetic fungicides to combat fungal diseases and prevent food losses. However, the widespread use of synthetic fungicides has led to the development of fungicide-resistant pathogens [216,217,218] and raised concerns about their impact on the environment and human health. BCAs have been proposed as an alternative, but their disease management capacity is often limited and dependent on unpredictable environmental factors. To achieve similar effectiveness as chemical fungicides, they must also be used in high doses, which often limits their application. Furthermore, the volatile and oxidizing nature of essential oils leads to high cost, which is also a significant limitation for large-scale use [207]. The integration of BCAs with fungicides is a promising approach to address these challenges, providing sustainable and effective solutions for managing plant diseases while mitigating the adverse effects of synthetic fungicides [189].

## 5. Integrated Control

Both biological control agents and plant extracts can constitute functional elements of an Integrated Pest Management System (IPM). Integrated Pest and Disease Management (IPDM) strategies have become increasingly essential in modern agriculture, driven by the need for sustainable practices that minimize the adverse effects of synthetic chemicals on both the environment and human health. While these integrated approaches hold great potential, further research is needed to optimize the timing and frequency of the application of BCAs and their compatibility with fungicides to maximize their impact on disease control and crop yield in various agricultural systems [219,220].

Integrating BCAs with fungicides can diversify antifungal treatments, reduce fungicide doses for disease management, and minimize residue on harvested crops. This approach also reduces the risk of pathogens developing resistance [221]. Numerous studies have explored the compatibility of various BCAs with fungicides across various crop systems. However, some experiments were carried out on fungicides that have now been withdrawn [222,223,224,225,226,227]. For instance, Sunkad et al. (2023) investigated the tolerance of *Trichoderma* species, including *Trichoderma asperellum*, against a range of fungicides [228]. The results revealed varying degrees of compatibility, with systemic fungicides such as azoxystrobin showing high compatibility with *Trichoderma*. The obtained fungicide concentrations inhibited all tested pathogenic strains without significantly inhibiting *Trichoderma* strains, the growth of which further enhanced the inhibitory effect of the fungicide-BCA combination. Similar studies have examined the compatibility of biocontrol yeasts *Rhodosporidium kratochvilovae* and *Cryptococcus laurentii*, and of bacteria *Pseudomonas syringae* with fungicides including boscalid, cyprodinil, fenhexamid, and thiabendazole. The results showed promise for controlling diseases including blue mold on apples [229,230].

Integrated approaches have also been developed and optimized for specific crop-disease systems. For example, in the case of tulsi wilt caused by *Fusarium oxysporum*, the integration of *Trichoderma* with the fungicides carbendazim and mancozeb has proven highly effective at reducing disease severity and enhancing plant growth [231]. This approach not only provides efficient disease management but also highlights the potential for eco-compatible agriculture. In the context of wheat diseases such as powdery mildew and *Fusarium* head blight (FHB), integrated approaches have been explored with BCAs including *Rhodosporidium kratochvilovae*, *Cryptococcus laurentii*, and *Aureobasidium pullulans*. These BCAs were combined with mineral salts, sulfur, and synthetic fungicides including azoxystrobin, tebuconazole, and tetraconazole. The results showed reduced disease severity, increased grain yield, and improved grain weight [232,233].

Integrated control does not end, however, with the combination of microorganisms and synthetic fungicides. Other approaches involve the combination of plant extracts/essential oils with fungicides, microorganisms, or organic compounds. In 2015, Fielding et al. (2015) examined the botryticidal effectiveness of South African medicinal plant extracts, both alone and with kresoxim-methyl fungicide [234]. Synergistic inhibitory effects were observed in vitro, and in vivo experiments on apples demonstrated synergistic and additive decay inhibition effects, suggesting the natural compounds in these plants enhance kresoxim-methyl efficacy [234]. Adandonon et al. (2006) investigated the efficacy of biocontrol agents, alone or combined with *Moringa oleifera* leaf extracts [235]. The results showed over 94% disease control in the greenhouse and 70% disease control in the field, with increases in yield. El-kazzaz et al. (2015) studied the efficacy of salicylic acid and plant extracts against rice kernel smut disease, recommending their inclusion in integrated pest management for combating *Tilletia barclayana* [236].

When describing integrated control, the topic of organic farming also cannot be ignored. Organic farming is a sustainable and environmentally friendly approach to agriculture that emphasizes the use of natural processes and inputs to grow crops. A core principle of organic farming is soil health [237]. Unlike conventional farming, which often relies on synthetic chemicals, organic farming focuses on maintaining ecological balance and enhancing biodiversity. Techniques such as crop rotation, green manure, cover cropping, and composting are adapted to maintain and improve soil quality and enhance its water retention capacity. To combat pests and diseases, organic farmers employ an integrated pest management (IPM) approach. It involves using a combination of biological, cultural, physical, and mechanical controls. In the case of fungal diseases, means such as crop rotation, breeding of resistant plant varieties, proper spacing, or natural fungicides are used. Crop rotation prevents build-up of pathogens in the soil by altering the crops that are susceptible to different diseases, while proper spacing reduces humidity by enhancing air circulation [238]. Natural fungicides include copper and sulfur-based products as well as biofungicides derived from beneficial microorganisms like *Trichoderma* or *Bacillus* species. Such practices allow for a system where chemical pesticides are not needed to control various dangers. However, while these methods are effective, it is not guaranteed that they will also work on large-scale farms, in the form they are employed in currently. Additionally, copper-containing fungicides might not be the perfect choice, as in his work Burandt et al. (2024) explains the potential risks of copper overuse from many different angles [239].

These studies collectively highlight the potential of plant extracts and organic compounds in combination with microorganisms or synthetic substances, as effective and environmentally friendly strategies for pest and disease management in agriculture. However, more work is needed to study the interactions of plant extracts with both fungicides and microorganisms exhibiting inhibitory capabilities against mold growth. Better understanding of these interactions would improve our ability to realistically assess the utility and applicative possibilities of integrated solutions in various agricultural areas.

Due to the lack of universal legislation, active substances may be allowed or prohibited in different parts of the world. This can create problems for comparison of experimental data. There is a need to keep the data as up to date as possible to ensure that future experiments will use substances that are permitted by the regional authorities, allowing for better data comparison between different microorganisms.

## 6. Summary

Synthetic (chemical) fungicides are currently the most common means for protecting plants and grains against phytopathogens. Farmers use them to protect their crops against the constant risk of infection with phytopathogens and, consequently, prevent economic losses. However, synthetic fungicides can also have a negative impact on the natural environment, including human and animal health. Moreover, when fungicides are overused, applied at sub-lethal doses, or used inappropriately, fungi can develop resistance to these chemicals. Concerns about their impact on human health, the environment, and the development of resistant pathogens have led to increased scrutiny and regulation. In response, many regions, including the European Union, have introduced restrictions on the use of synthetic fungicides.

Biological control agents appear to be a safe alternative to chemical fungicides for plant and seed protection. Biological control agents can help reduce the reliance on chemical fungicides. However, the complete or partial elimination of synthetic fungicide usage would create a gap that is difficult to fill in the short term. Furthermore, natural fungicides also have some disadvantages (Table 4). The choice between synthetic and natural fungicides for plant and seed treatments depends on many factors, including the type of crop, environmental conditions, and local regulations governing the use of plant protection products. It is important to strike a balance between producing efficient crops and protecting the environment and human health.

In summary, the choice of preparations used to protect plants in agriculture is not always simple or straightforward. A promising approach seems to be the appropriate integration of natural and synthetic agents. Such integrated strategies can help to provide an effective solution to plant disease control problems while mitigating the adverse effects of synthetic fungicides alone. It is important to ensure that synthetic fungicides are used effectively so that they can play a part in more sustainable agriculture. To fully realize the potential of integrated approaches in responsible agricultural management, further investment and development in the following areas are necessary:

- Registration procedures and regulatory guidelines;
- Research tools that monitor and evaluate the risk of long-term use, in terms of maintaining the biodiversity of natural environments and, above all, the safety of people and animals.

With further development of technology, solutions applied directly to plants might soon be complemented by advanced approaches such as precision agriculture (or precision farming). This method utilizes remote sensing, yield monitoring, robotics, machine learning, and data analysis to boost efficiency and productivity in agriculture. Through data analysis and machine learning, the usage of fertilizers, pesticides, and water can be optimized, thereby reducing the overall costs of food production [240]. Additionally, collected data could become a valuable source of information for developing highly effective Integrated Pest Management (IPM) strategies [241]. Fungicides, which are crucial for controlling fungal diseases in crops, fit seamlessly into the framework of precision agriculture. The integration of fungicides into this advanced agricultural approach can lead to solutions such as the targeted application of fungicides (e.g., using the fungicide in the areas of the field that are most prone to infections) or applying the fungicide at optimal times to reduce the need for blanket applications, which could further reduce their use. The integration of fungicides into precision agriculture will likely enhance their effectiveness while reducing environmental and economic costs. By overcoming current hurdles such as high costs, technology integration, and education, precision agriculture, coupled with innovative fungicide developments, promises a more sustainable and productive future for farming [242,243].

## Figures and Tables

**Table 1:** Annual cereal production and pesticide use by the largest global food producers [28,30,31].

Region	Annual Production of Cereals * [million tonnes]	Crop Area * [million ha]	Estimated Pesticide Usage [kg/ha]	Estimated Fungicide Usage [kg/ha]
World	2166	482.5	117.9	48.4
EU (27)	254.3	47.5	38.8 (EU)	19.4 (EU)
US	398.6	47.9	4.3	0.3
Brazil	121.2	24.9	12.7	2.8
China	418.2	67.5	1.4	0.4

* Wheat, rye, barley, oats, corn.

**Table 2:** Examples of fungicides commonly used in cereal protection and their regulatory status in selected regions [35,61,62,63,64,65,66].

Fungicide	Group Name (FRAC Group)	Examples of Application Area	Permitted Usage in Agriculture EU/USA/CHINA/BRAZIL	References
Morpholines				
Tridemorph	Amines (5)	Foliar spraying	−/+/+/−	[67]
Pyrimidines				
Cyprodinil	AP-fungicides (9)	Foliar spraying, soil fumigant	+/+/+/+	[68,69]
Carboxylic Acid Esters				
Ethyl formate	Carboxylic Acid Esters (n.a.)	Grain fumigant	−/?/?/?	[70]
Phthalonitriles				
Chlorothalonil	Chloronitriles (M 05)	Foliar spraying	−/+/+/+	[71]
Dicarboximides				
Iprodione	Dicarboximides (2)	Seed treatment, foliar spraying	−/+/+/+	[72,73]
Dithiocarbamates				
Mancozeb	Dithiocarbamates (M 03)	Seed treatment	−/+/+/+	[74,75]
Metam-sodium		Soil fumigation	+/+/+/+	[76]
Thiram		Foliar spraying	−/+/+/+	[77]
Imidazoles, Triazoles				
Bitertanol	DMIs (3)	Foliar spraying, seed treatment	−/+/+/−	[78]
Cyproconazole		Foliar spraying	−/+/−/+	[79]
Difenoconazole		Seed treatment	+/+/+/+	[80,81]
Imazalil		Seed treatment	+/+/+/+	[82]
Myclobutanil		Seed treatment, foliar spraying	−/+/+/+	[83]
Prochloraz		Seed treatment, foliar spraying	−/+/+/−	[84]
Propiconazole		Seed treatment, foliar spraying	−/+/+/+	[85]
Tebuconazole		Foliar spraying	+/+/+/+	[86,87]
Triadimefon		Seed treatment	−/+/+/+	[88]
Triadimenol		Foliar spraying	−/+/+/+	[89]
Triticonazole		Seed treatment	+/+/+/+	[90,91]
Epoxides				
Ethylene oxide	Cyclic ethers (n.a.)	Grain fumigant	−/−/−/−	[92]
1,2,4-thiadiazoles				
Etridiazole	Heteroaromatics (32)	Seed treatment	−/+/−/+	[93]
Inorganic compounds				
Aluminum phosphide	Aluminum, Copper salts etc. (NC)	Grain fumigant	+/+/−/?	[94,95,96]
Copper oxychloride		Foliar spraying, seed treatment	+/+/+/+	[97]
Copper sulfate		Seed treatment	+/+/−/+	[98]
Benzimidazoles				
Benomyl	MBCs (1)	Seed treatment, foliar spraying	−/−/+/−	[99,100]
Carbendazim		Foliar spraying, Seed treatment	−/+/+/+	[101]
Thiabendazole		Seed treatment	+/+/+/+	[102]
Thiophanates				
Thiophanate-methyl	MBCs (1)	Seed treatment	−/+/+/+	[103]
Halogenated aliphatics				
Ethylene dibromide	Organobromine compounds (n.a.)	Grain fumigant	−/−/−/−	[104]
Methyl bromide		Soil fumigation	−/−/−/−	[105]
Chloropicrin	Organochlorine compounds (n.a.)	Soil fumigant, grain fumigant	−/+/−/?	[106,107]
1,3-dichloropropene		Soil fumigant	−/+/+/+	[108,109]
Ethylene dichloride		Grain fumigant	−/−/−/−	[110]
Acylalanines				
Metalaxyl	Phenylamides (4)	Seed treatment	+/+/−/+	[111]
Phthalimides				
Captafol	Phthalimides (M 04)	Seed treatment	−/−/−/−	[112,113]
Captan		Soil fumigant, seed treatment	+/+/+/+	[114,115]
Strobilurins				
Azoxystrobin	QoIs (11)	Foliar spraying	+/+/+/+	[99,116,117]
Trifloxystrobin		Foliar spraying	+/+/+/+	[117]
Kresoxim methyl		Foliar spraying	+/+/+/+	[118]
Pyraclostrobin		Foliar spraying	−/+/+/+	[119]
Oxazoles				
Famoxadone	QoIs (11)	Foliar spraying	+/+/+/+	[120]
Oxathiins				
Carboxin	SDHIs (7)	Seed treatment	−/+/+/+	[121]
Oxycarboxin		Seed treatment, foliar spraying	−/+/+/+	[122]

(+) Permitted, (−) Not permitted; (?) No data, n.a.—information unavailable.

**Table 3:** Microorganisms as biocontrol agents.

Microorganisms	Pathogens	Active Compounds	References
Bacteria			
*Bacillus subtilis*	*Fusarium graminearum*	surfactin, iturin, fengycin lipopeptides	[151,152,154]
*Bacillus megaterium*	*Fusarium graminearum*	not studied	[154]
*Bacillus vallismortis*	*Fusarium graminearum* *Alternaria alternata* *Rhizoctonia solani Cryphonectria parasitica* *Phytophthora capsici*	bacillomycin D	[155]
*Bacillus amyloliquefaciens*	*F. graminearum* *Fusarium* spp.	iturin lipopeptide	[157,165]
*Paenibacillus polymyxa*	*F. graminearum*	not studied	[166]
*Lacticaseibacillus rhamnosus*	*F. graminearum*	not studied	[158]
*Lactiplantibacillus plantarum*	*F. graminearum* *Fusarium* spp.	not studied	[157,158]
*Pseudomonas fluorescens*	*F. graminearum*	not studied	[167]
*Pseudomonas chlororaphis*	*Drechslera graminea* *D. teres* *D. avenae* *Ustilago avenae* *U. hordei* *Tilletia caries*		[168]
*Streptomyces* spp.	*F. graminearum* *Rhizoctonia solani*	secondary metabolites chitinases volatile organic compounds	[150,167,168,169]
*Azotobacter nigricans*	*F. sporotrichioides* *F. graminearum* *F. poae* *F. crookwellense* *F. equiseti* *F. sambucinum* *F. culmorum*	not studied	[170]
Yeasts			
*Sporidiobolus pararoseus*	*Fusarium fujikuroi*	not studied	[161]
*Pichia guilliermondii*	*Fusarium fujikuroi* *Penicillium roqueforti*	not studied	[161,162]
*Metschnikowia pulcherrima*	*Fusarium fujikuroi*	not studied	[161]
*Cryptococcus flavescens*	*F. graminearum*	not studied	[171]
*Pichia anomala*	*Penicillium roqueforti*	not studied	[162]
*Pichia burtonii*			
*Pichia farinosa*			
*Pichia membranifaciens*			
*Candida silvicola*			
*Candida fennica*			
*Candida pelliculosa*			
*Candida silvicultrix*			
Molds			
*T. asperellum*	*F. graminearum* *Pseudomonas syringae*	not studied induced resistance	[172] [173]
*T. citrinoviride*	*F. graminearum*	not studied	[158]
*T. harzianum*	*F. verticillioides* *F. graminearum* *F. oxysporum* *B. cinerea*	secretion of chitinase, competition for space	[171,172,173,174]
*T. brevicrasum*	*Rizoctonia solani*	mycoparasitism	[173]
*Clonostachys rosea*	*F. culmorum* *F. graminearum* *F. verticillioides* *F. crookwellense* *Alternaria dauci* *A. radicina* *Botrytis cinerea* *B. aclada* *Bipolaris sorokiniana* *Drechslera teres* *Helminthosporium solani* *Moniliophthora roreri*, *Phytophthora palmivora* *Rhizoctonia solani* *Rhynchosporium communea* *Sclerotinia sclerotiorum*	secretion cell-wall-degrading enzymes	[163,164]

**Table 4:** Summary of advantages and disadvantages of natural and synthetic fungicides.

Antifungals	Advantages	Disadvantages
Natural	more environment friendly, decompose more easily in the natural environment and may have less impact on ecosystems; fewer residues in plants and food products; lower risk of developing resistance.	often shorter duration of action than synthetic substances, more frequent treatments required; if used incorrectly they may be toxic to humans and animals; less stable composition.
Synthetic	often more effective in plant protection and more efficient for plant production; their composition and properties can be precisely controlled; more stable and durable, their effect lasts longer than that of natural substances; better control of seed dressing processes and more precisely adjusted dosing; cheaper to produce, easier to store and transport.	often toxic to humans and animals, health risks; negative impact on the environment, contamination of soil, groundwater, and surface water.

## Data Availability

Not applicable.
